# Tumidulin, a Lichen Secondary Metabolite, Decreases the Stemness Potential of Colorectal Cancer Cells

**DOI:** 10.3390/molecules23112968

**Published:** 2018-11-14

**Authors:** Yi Yang, Suresh R. Bhosle, Young Hyun Yu, So-Yeon Park, Rui Zhou, İsa Taş, Chathurika D. B. Gamage, Kyung Keun Kim, Iris Pereira, Jae-Seoun Hur, Hyung-Ho Ha, Hangun Kim

**Affiliations:** 1 College of Pharmacy, Sunchon National University, 255 Jungang-ro, Sunchon, Jeonnam 57922, Korea; yangyi_520@hotmail.com (Y.Y.); bhoslesuresh1005@gmail.com (S.R.B.); chmyyh@gmail.com (Y.H.Y.); sinbu17@naver.com (S.-Y.P.); zhourui274@hotmail.com (R.Z.); mr.isatas@gmail.com (İ.T.); chathurika.gamage@gmail.com (C.D.B.G.); 2 Korean Lichen Res. Institute, Sunchon National University, 255 Jungang-ro, Sunchon, Jeonnam 57922, Korea; 3 Medical Research Center for Gene Regulation, Brain Korea 21 Project, Chonnam National University Medical School, 160 Baekseo-ro, Dong-gu, Gwangju 61469, Korea; kimkk@chonnam.ac.kr; 4 Institute of Biological Sciences, Universidad de Talca, Talca 747-721, Chile; ipereira@utalca.cl

**Keywords:** lichen, secondary metabolites, tumidulin, stemness potential, colorectal cancer cells, oncogene, transcriptional regulation

## Abstract

Lichens produce various unique chemicals that are used in the pharmaceutical industry. To screen for novel lichen secondary metabolites that inhibit the stemness potential of colorectal cancer cells, we tested acetone extracts of 11 lichen samples collected in Chile. Tumidulin, isolated from *Niebla* sp., reduced spheroid formation in CSC221, DLD1, and HT29 cells. In addition, mRNA expressions and protein levels of cancer stem markers aldehyde dehydrogenase-1 (ALDH1), cluster of differentiation 133 (CD133), CD44, Lgr5, and Musashi-1 were reduced after tumidulin treatment. Tumidulin decreased the transcriptional activity of the glioma-associated oncogene homolog zinc finger protein (Gli) promoter in reporter assays, and western blotting confirmed decreased Gli1, Gli2, and Smoothened (SMO) protein levels. Moreover, the tumidulin activity was not observed in the presence of Gli and SMO inhibitors. Together, these results demonstrate for the first time that tumidulin is a potent inhibitor of colorectal cancer cell stemness.

## 1. Introduction

Colorectal cancer (CRC) is one of the most commonly diagnosed cancers worldwide [1], and its high mortality rate has made it a priority in clinical cancer therapy [2]. Large numbers of CRC patients present with metastatic disease [3], and the 5-year survival rate is only 12.5% [3]. Furthermore, resistance to chemotherapy occurs in 90% of patients with metastatic cancer, and this is believed to be the main reason for treatment failure, despite the development of numerous therapeutic agents targeting CRC in the past decade [4]. Mounting evidence indicates that cancer stem cells (CSCs) contribute to chemotherapy resistance [5,6,7], and recent studies suggest that the efficiency of chemotherapy could be improved by exploiting the therapeutic opportunities provided by the molecular properties of CSCs. Cluster of differentiation (CD) 133 [8,9], CD44 [10], and leucine-rich repeat-containing G-protein coupled receptor 5 (LGR5) [11] are colorectal CSC markers related to proliferation, invasion, metastasis, and chemoresistance, and aldehyde dehydrogenase (ALDH) activity has been linked to the mechanism of chemoresistance in CSCs [12]. Therefore, it is important to seek new therapeutic strategies targeting CSC-like properties.

The extensive chemical diversity of lichen secondary metabolites makes them a powerful natural resource for candidate pharmaceutical reagents. In a previous study, we described the wide pharmaceutical application of lichen bioresources in anti-cancer chemotherapy reagent screening [13,14,15,16]. However, no attempt has been made to screen anti-cancer activity based on CSC-like traits, which has the potential to identify more efficient chemotherapeutic agents. Therefore, in the present work, we examined the inhibitory activity of 11 lichen species from Chile against the stemness potential of CRC cells, and investigated the possible molecular mechanisms underlying this inhibitory activity to identify novel compounds that could lower the chemoresistance of target therapeutic drugs.

## 2. Results

### 2.1. Acetone Extracts of Lichens Collected in Chile Inhibit CRC Cell Stemness

**Figure 1:**
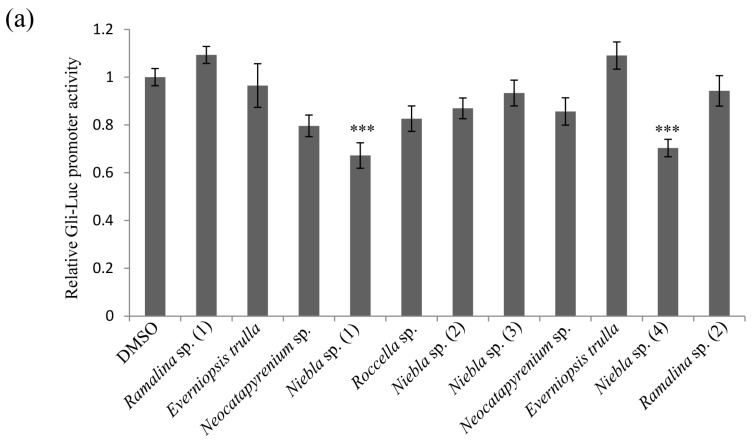

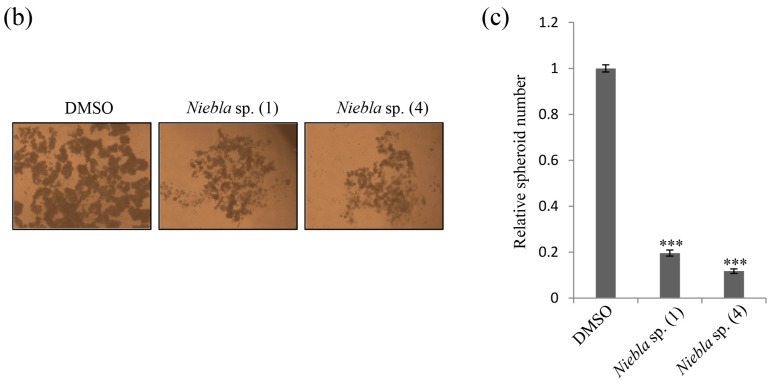
Acetone extracts of lichens collected in Chile decrease CSC221 cell stemness. (**a**) Quantitative analysis of Gli-luc reporter assays of NIH 3T3 cells (stably incorporating Gli-dependent firefly luciferase and constitutive Renilla luciferase reporters) treated with 5 μg/mL acetone extracts of *Ramalina* sp. (1), *Everniopsis trulla*, *Neocatapyrenium* sp., *Niebla* sp. (1), *Roccella* sp., *Niebla* sp. (2), *Niebla* sp. (3), *Neocatapyrenium* sp., *Everniopsis trulla*, *Niebla* sp. (4), and *Ramalina* sp. (2) for 48 h. (**b**) Representative images of spheroid formation of CSC221 cells treated with extracts of *Niebla* sp. (1) or *Niebla* sp. (4) for 14 days. (**c**) Quantitative analysis of the number of spheroids following each treatment. Quantitative data were obtained from three independent experiments (*n* = 3). Data represent mean ± standard error of the mean (SEM), and analysis was performed by one-way ANOVA. *** *p* < 0.001 compared with dimethyl sulfoxide (DMSO)-treated CSC221 cells.

The self-renewing ability of cancer stem cells (CSCs) can result in resistance to targeted therapies. To identify inhibitory substances among lichen secondary metabolites, Gli luciferase activity was tested against 11 acetone extracts of Chilean lichen thalli for in the NIH 3T3 cell line in which a Gli reporter gene was stably incorporated. For this, 5 µg/mL concentration of extracts was tested as cytotoxicity was not observed at this concentration (Figure S1). As shown in Figure 1a, *Niebla* sp. (1) and *Niebla* sp. (4) both inhibited Gli-luc activity at a concentration of 5 µg/mL. To further check whether the above lichen extracts possessed inhibitory activity against the spheroid formation ability of CSC221 cells (human colorectal adenocarcinoma-enriched cancer stem cells [17]), spheroid formation assays were performed using ultra-low attachment 24-well plates with serum-free medium. As shown in Figure 1b, the number of spheroids in cells treated with acetone extracts was lower than in dimethyl sulfoxide (DMSO)-treated controls, and quantitative analysis revealed that the differences were significant (Figure 1c). These results showed that acetone extracts of *Niebla* sp. (1) and *Niebla* sp. (4) exhibited inhibitory activity against colorectal cancer (CRC) cell stemness.

### 2.2. Tumidulin, A Lichen Secondary Metabolite from Niebla sp., Inhibits CRC Cell Stemness

**Figure 2:**
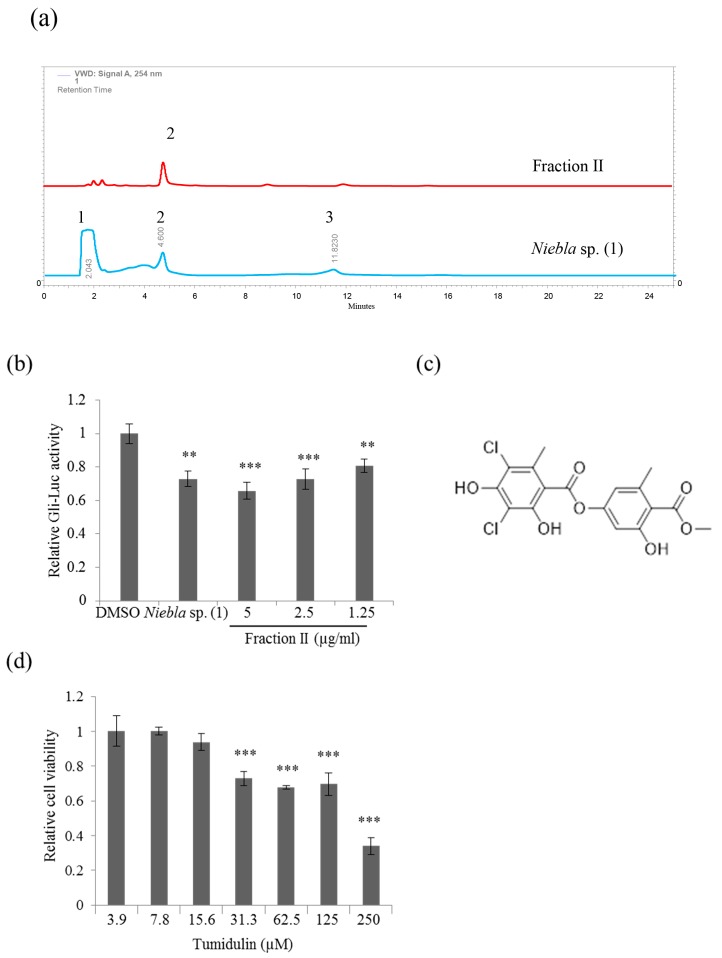
Tumidulin is an active lichen secondary metabolite from *Niebla* sp. (1) that inhibits CSC221 cell stemness. (**a**) High-performance liquid chromatography (HPLC) analysis of the inhibitory activity of stemness in CSC221 cells by crude and active *Niebla* sp. (1) fractions using a methanol:water:phosphoric acid (80:20:1, *v*/*v*/*v*) solvent system. (**b**) Quantitative analysis of reporter assays of NIH 3T3 cells treated with a 5 μg/mL acetone extract of *Niebla* sp. and various concentrations of the active (tumidulin) fraction for 48 h. (**c**) Chemical structure of tumidulin. (**d**) Relative viability of CSC221 cells treated with tumidulin for 48 h by MTT assay. Quantitative data were obtained from three independent experiments (*n* = 3). Data represent mean ± SEM, and analysis was performed by one-way ANOVA. ** *p* < 0.01 and *** *p* < 0.001 compared with DMSO-treated CSC 221 cells.

The chemical compounds isolated from *Niebla* sp. (1; CH130494) and *Niebla* sp. (4; CH130414) were almost identical according to the results of thin layer chromatography (TLC) (Figure S2); hence *Niebla* sp. (1) was chosen for subsequent research. To investigate the active compounds possessing inhibitory activity against CRC cell stemness, an acetone extract of *Niebla* sp. (1) was analyzed by high-performance liquid chromatography (HPLC) (Figure 2a), and the three main fractions were collected and tested in Gli-luc reporter assays using NIH 3T3 cell lines. The fraction II decreased Gli-luc activity in a dose-dependent manner comparable to crude extract (Figure 2b). This active fraction was therefore used for purification and structural identification of active components. The active, purified fraction was confirmed as tumidulin (molecular weight = 401.192 g/mol, purity > 99%) by liquid chromatography-mass spectrometry (LC-MS) and nuclear magnetic resonance (NMR) analyses (Figure 2c; Figures S3–S7 in File S1). The 3-(4,5-dimethylthiazol-2-yl)-2,5-diphenyltetrazolium bromide (MTT) assay revealed that cell viability was not significantly affected by tumidulin on CSC221 cells at less than 5 µg/mL (= 12.5 µM) concentration (Figure 2d). These results indicate that tumidulin is the active compound responsible for the inhibitory activity against CRC cell stemness in the *Niebla* sp. (1) acetone extract.

### 2.3. Tumidulin Inhibits Spheroid Formation in CRC Cells

**Figure 3:**
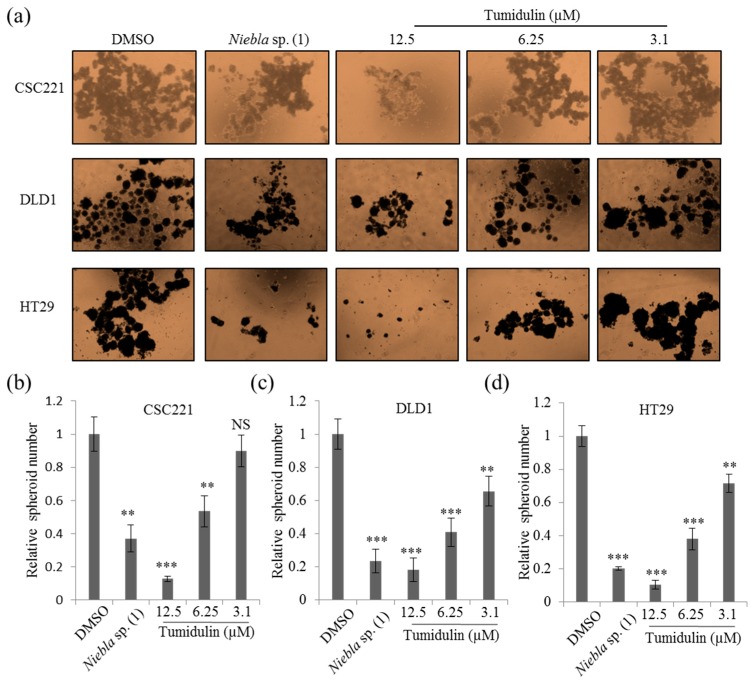
Effects of crude acetone extracts of *Niebla* sp. (1) and tumidulin on spheroid formation of colorectal cancer (CRC) cells. CRC cells were treated with crude extracts of *Niebla* sp. (1) and various concentrations of tumidulin. After 14 days incubation, spheroid formation of CRC cells was calculated as described in the Materials and Methods. (**a**) Representative images of spheroid formation of CSC221, DLD1, and HT29 cells following treatment with crude extracts of *Niebla* sp. (1) and the purified tumidulin fraction at the indicated concentrations. (**b**–**d**) Quantitative spheroid assay data following treatment of CSC221 (**b**), DLD1 (**c**), and HT29 (**d**) cells with crude extracts of *Niebla* sp. (1) and the purified tumidulin fraction. Quantitative data were obtained from three independent experiments (*n* = 3). Data represent mean ± SEM, and analysis was performed by one-way ANOVA. ** *p* < 0.01; *** *p* < 0.001; NS: no significant difference compared with DMSO-treated CRC cells.

To further confirm the CRC cell stemness inhibitory activity of tumidulin, the reduction in cancer stemness by acetone crude extracts of *Niebla* sp. (1) and tumidulin was evaluated by measuring spheroid formation in various CRC cell lines. As shown in Figure 3a, the number of spheroids following treatment with a 5 μg/mL crude extract of *Niebla* sp. (1) and various concentrations of tumidulin was significantly less than in DMSO-treated control cells for all CRC cell lines tested, including CSC221, DLD1, and HT29 cells. However, their profiles at the same concentrations exhibited some differences. Tumidulin did not inhibit spheroid formation in CSC221 cells at 1.25 µg/mL (= 3.1 µM), but it did decrease the spheroid number in the other two CRC cell lines (DLD1 and HT29). Quantitative analysis confirmed that tumidulin inhibited spheroid formation in a dose-dependent manner for all three CRC cells (EC_50 CSC221_ = 2.609 µg/mL = 6.523 µM; EC_50 DLD1_ = 1.926 µg/mL = 4.815 µM; EC_50 HT29_ = 1.944 µg/mL = 4.860 µM) (Figure 3b,c). It should be noted that cell viability was not significantly affected by acetone extracts of *Niebla* sp. (1) and tumidulin on tested CRC cells at 12.5 µM (= 5 µg/mL) concentration (Figure S8), suggesting that cell death or blockade of cell proliferation is not involved in these effects. Taken together, these results indicate that tumidulin decreased the stemness of CRC cells.

### 2.4. Tumidulin Decreases Cancer Stem Markers

**Figure 4:**
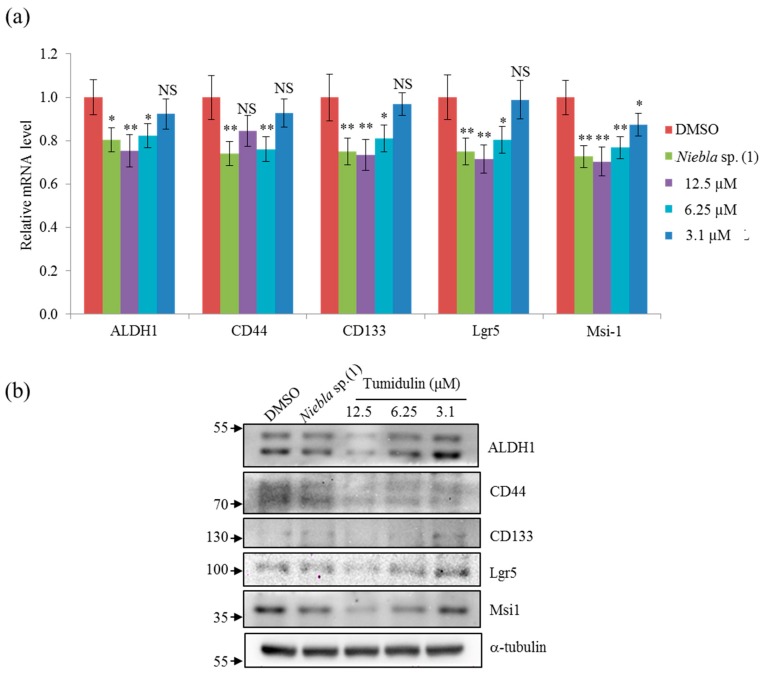
Effects of crude extracts of *Niebla* sp. (1) and tumidulin on cancer stemness-related gene expression and protein level. CSC221 cells were treated with 5 μg/mL acetone extracts of *Niebla* sp. (1) or various concentrations of tumidulin for 48 h. (**a**) Quantitative analysis of mRNA expression levels of cancer stem markers aldehyde dehydrigebase-1 (ALDH1), cluster of differentiation 133 (CD133), CD44, Lgr5, and Musashi-1. Data represent mean ± SEM, and analysis was performed by one-way ANOVA. * *p* < 0.05; ** *p* < 0.01; NS: no significant difference compared with DMSO-treated CSC221 cells. (**b**) Representative immunoblots are shown with the indicated antibodies.

ALDH1, CD133, CD44, Lgr5, and Msi-1 are markers for the acquisition of cancer stemness. Since tumidulin inhibited spheroid formation in CRC cells (Figure 3), we hypothesized that the expression of CSC markers could be downregulated at compound concentrations at which spheroid formation was reduced. To investigate whether tumidulin affected the expression of cancer stemness markers in CRC cells, mRNA expressions and protein levels of ALDH1, CD133, CD44, Lgr5, and Msi-1 were measured by qRT-PCR and western blotting, respectively. As shown in Figure 4a,b, the inhibitory activity of tumidulin was similar to that of the crude acetone extract of *Niebla* sp. (1), and it reduced mRNA expressions and protein levels of all five cancer stem markers at concentrations of 2.5 and 5 μg/mL (= 6.25 and 12.5 µM).

### 2.5. Tumidulin Reduces Gli and SMO Protein Levels

**Figure 5:**
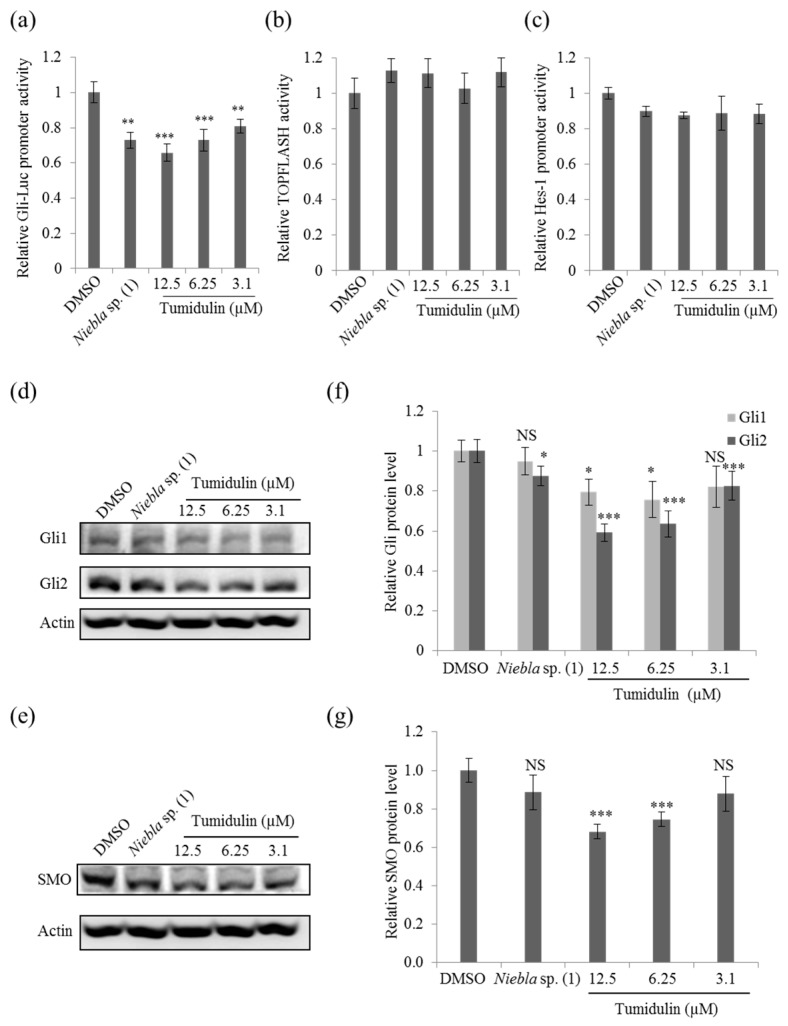
Effects of crude acetone extracts of *Niebla* sp. (1) and tumidulin on transcription factor activity and the expression of proteins related to cancer stemness. (**a**–**c**) Relative activities of promoters related to Hedgehog, Wnt, and Notch signaling pathways. HEK293T cells were co-transfected with the pRL-TK (Renilla) plasmid and the pGli-luc (**a**), pTOPFLASH (**b**), and pHES-luc (**c**) reporter plasmids (firefly). After 12 h, transfected cells were treated with crude extracts of *Niebla* sp. (1) or various concentrations of tumidulin and incubated for an additional 48 h. The relative firefly luciferase activity of treated vs. control groups is shown. Quantitative data were obtained from at least two independent experiments. (**d**–**g**) CSC221 cells were treated with 5 μg/mL *Niebla* sp. (1) or various concentrations of tumidulin. After incubation for 48 h, cells were lysed and total protein was subjected to immunoblot analyses with the indicated antibodies. (**d**,**e**) Representative immunoblots of Gli1/2 (**d**) and SMO (**e**). (**f**,**g**) Relative protein levels of Gli1/2 (**f**) and SMO (**g**) compared with the untreated group. Data represent the mean ± SEM, and analysis was performed by one-way ANOVA. * *p* < 0.05; ** *p* < 0.01; *** *p* < 0.001; NS: no significant difference compared with DMSO-treated CSC221 cells.

To identify target signaling pathways involved in the inhibition of cancer stemness by tumidulin, reporter assays were performed using the HEK293T cell line transfected with Gli-luc, TOPFLASH-luc, and Hes-1-luc genes conjugated with firefly plasmids. Based on the relative values calculated with Renilla luciferase as an internal control, only Gli-luc activity was downregulated significantly by tumidulin treatment in a dose-dependent manner (Figure 5a–c). Since Gli is the main transcription factor in the sonic hedgehog (SHH) signaling pathway, this pathway is likely involved in the reduction of CRC stemness by tumidulin treatment. These results suggest that tumidulin might decrease CRC cancer stemness by downregulating the expression of CSC markers and inhibiting Gli transcription.

To confirm the involvement of the SHH signaling pathway in the CRC stemness reduction activity of tumidulin, Gli1/2, and smoothened (SMO) protein levels were measured. As shown in Figure 5d,f, both Gli1 and Gli2 protein levels were significantly downregulated by tumidulin at a concentration of 2.5 or 5 μg/mL (= 6.25 or 12.5 µM) in the CSC221 cell line. The Gli2 protein was decreased to a greater extent than Gli1 based on the quantification results shown in Figure 5f. Furthermore, SMO protein levels were also reduced by tumidulin treatment at 2.5 or 5 μg/mL (= 6.25 or 12.5 µM) (Figure 5e,g). These Gli proteins are the terminal effectors of SHH signaling, and SMO is a transcriptional regulator that acts upstream of the canonical hedgehog (Hh) pathway [18]. Together, these results suggest that tumidulin inhibits the Hh signaling pathway by suppressing Gli and SMO.

### 2.6. Tumidulin Activity on Reducing ALDH1 Expression is Gli and SMO Dependent

**Figure 6:**
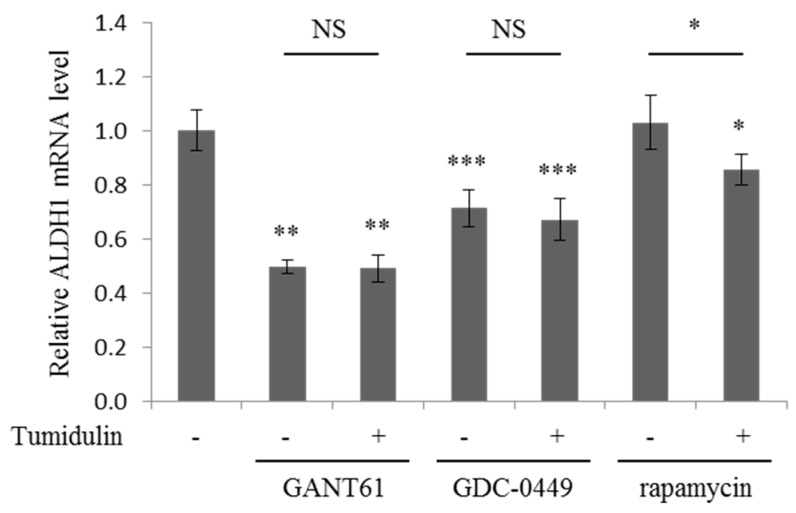
Effects of tumidulin on mRNA expression levels of ALDH1 in the presence of Gli, SMO, and mTOR inhibitors. CSC221 cells were treated with 5 μg/mL GANT61 (Gli inhibitor), GDC-0449 (SMO inhibitor), and rapamycin (mTOR inhibitor) together with or without 12.5 µM tumidulin for 48 h. Quantitative analysis of mRNA expression level of ALDH1 were presented as mean ± SEM, **(***n* = 3). Analysis was performed by one-way ANOVA. * *p* < 0.05; ** *p* < 0.01; *** *p* < 0.001; NS: no significant difference compared with DMSO-treated CSC221 cells or between indicated groups.

To confirm the involvement of the Gli and SMO in the CRC stemness reduction activity of tumidulin, the mRNA expression level of ALDH1 were measured in the presence of Gli and SMO inhibitors [18]. As shown in Figure 6, Gli inhibitor (GANT61) and SMO inhibitor (GDC-0449) treatment decreased the mRNA expression of ALDH1, and GANT61 which targets downstream of pathway reduced the mRNA expression more than those of GDC-0449. In the presence of these inhibitors, tumidulin did not further decrease the ALDH1 expression, suggesting that the activity of tumidulin is dependent on Gli and SMO (Figure 6). Given the extent of reduction in ALDH1 expression (Figure 4), tumidulin seems to act upstream of the pathway. mTOR inhibition by rapamycin has been reported to decreased pancreatic cancer stem cell population [19]. However, treatment of rapamycin did not affect the ALDH1 expression in CSC221 cells, and tumidulin decreased the ALDH1 expression in the presence of rapamycin (Figure 6), suggesting that the activity of tumidulin is independent on mTOR pathway. Together, these results showed that the lichen secondary metabolite tumidulin decreases CRC cell stemness by inhibiting the Hh signaling pathway, and has great potential for therapeutics development.

## 3. Discussion

Numerous novel chemicals isolated from lichens display anti-cancer activity and show great potential for pharmaceutical usage. In the present study, the active chemical compound tumidulin was isolated from the lichen *Niebla* sp. from Chile, and found to exhibit inhibitory activity against CRC cell stemness. Tumidulin, also known as methyl 3,5-dichlorolecanorate, was first discovered in *Ramalina ceruchis* var. *tumidula* [20,21]. However, surprisingly, this compound could not be found in the type specimen, and as previously reported, many specimens of *Vermilacinia* were erroneously claimed to contain methyl 3,5-dichlorolecanorate [22]. In the present work, we isolated tumidulin from the lichen *Niebla* sp., and found that (1) this lichen secondary metabolite displays inhibitory activity against CRC stemness, (2) mRNA levels of several cancer stem markers including ALDH1, CD133, CD44, Lgr5, and Musashi-1 are downregulated by tumidulin treatment, and (3) the Hh signaling pathway is involved in the suppression of Gli and SMO protein levels by tumidulin treatment. These results indicate that tumidulin inhibits CRC stemness and has great pharmaceutical potential.

Since tumidulin was confirmed to decrease CRC cell stemness, several major signaling pathways related to this phenomenon, including Gli-mediated Hh, β-catenin-mediated Wnt, and Hes-1-mediated Notch signaling, were investigated using reporter assays. The Gli, TOPFLASH, and Hes-1 promoters were tested using firefly luciferase gene-conjugated plasmid transfection, and only Gli-luc activity was downregulated by tumidulin treatment, suggesting that Hh signaling may be involved in the inhibition of CRC stemness by tumidulin treatment. The Hh signaling pathway is a major regulator of cell differentiation, cell proliferation, and tissue polarity, and aberrant activation of the Hh signaling pathway is involved in various types of human cancers [18]. The gene encoding Gli was the first Hh signaling pathway gene found to be upregulated in human glioblastoma and derivative cell lines [23]. In the present study, Gli1/2 and SMO protein levels were downregulated following tumidulin treatment, which further confirmed that tumidulin decreases CRC cell stemness via suppression of the Hh signaling pathway. However, our results cannot determine whether other signaling pathways are involved in the downregulation of Gli protein expression, since Gli can be activated via both Hh-dependent and Hh-independent signaling pathways [18]. Thus, the exact molecular mechanisms require further investigation.

Together, the results of the present study suggest that tumidulin has great potential for development of a CRC cell stemness inhibitor, and further demonstrate the wide application of lichen bioresources for pharmaceutical reagent screening.

## 4. Materials and Method

### 4.1. Preparation of Lichen Extracts

Thalli of lichens were collected in 2013 during field trips in the National Park of Torres Del Paine, Patagonia, Chile, organized by Dr. Pereira from Talca University, Talca, Chile. The permit to collect lichen specimens from this location was issued by the Administration of the National Forestry Corporation (CONAF) of Punta Arenas, and the Administration of the National Park Torres del Paine, Magallanes Region and Chilean Antarctic, which is part of the National System of Protected Wild Areas of the State of Chile. Field studies did not involve any endangered or protected species. Duplicate samples have been deposited at the Korean Lichen and Allied Bioresource Center (KOLABIC) in the Korean Lichen Res. Institute (KoLRI), Sunchon National University, Korea. The dried thalli of the lichens were extracted with acetone then filtered and dried in rotary vacuum evaporator at 45 °C (Heidolph Instruments GmBH & CO, Schwabach, Germany). The dry extracts were dissolved in dimethyl sulfoxide (DMSO, Sigma-Aldrich, St. Louis, MO, USA) for further experiment use.

### 4.2. High-Performance Liquid Chromatography (HPLC) Analysis of Lichen Material

Acetone extracts of lichen thalli at a concentration of 5 mg/mL were subjected to high-performance liquid chromatography (HPLC) analysis using an Agilent 1260 instrument (Agilent Technology, Santa Clara, CA, USA) equipped with a YMC-Pack ODS reversed-phase column A (150 × 4.6 mm internal diameter) containing fully end-capped C18 material (particle size = 5 µm, pore size = 12 nm). Elution was performed at a flow rate of 1 mL/min with a solvent system of methanol:water:phosphoric acid (80:20:1, *v*/*v*/*v*) at a column temperature of 40 °C. Separation was monitored using an Agilent 1260 Infinity II Variable Wavelength Detector (1260 VWD; Agilent Technology) over a range of 190–800 nm. Observed peaks were detected at 254 nm. The sample injection volume was 50 µL. Salazinic acid (t_R_ = 2.27 ± 0.2 min) isolated from the lichen *Lobaria pulmonaria* was used as a standard, and voucher specimens were deposited in the herbarium of KOLABIC at the KoLRI, Sunchon National University, Korea.

### 4.3. Separation and Identification of Tumidulin

*Niebla* sp. (1; CL130494) extract was separated by HPLC as described above, and the fraction identified as tumidulin was collected. The supernatant was dried and weighed after centrifugation, and the HPLC assay was performed to confirm that the purified sample yielded a single peak. The structure of tumidulin was then confirmed by liquid chromatography-mass spectrometry (LC-MS, Shimadzu Corporation, Kyoto, Japan; Figure S2) and nuclear magnetic resonance (NMR, JEOL Ltd., Tokyo, Japan) analysis (Figures S3 and S4 in File S1).

### 4.4. Cell Culture and Reagents

The human colon cancer cell lines CSC221 (human colorectal adenocarcinoma-enriched CSCs [17]), DLD1, HT29 (CRC cells), and HEK293T (human embryonic kidney cells) were used in this study. Cells were cultured in Dulbecco’s modified Eagle’s medium (DMEM) supplemented with 10% fetal bovine serum and 1% penicillin-streptomycin solution under a humidified 5% CO_2_ atmosphere at 37 °C in an incubator. GDC-0449 (Vismodegib, A10258) was purchased from AdooQ Bioscience (Irvine, CA, USA). GANT61 (G9048) and rapamycin (R8781) were purchased from Sigma-Aldrich.

### 4.5. Reporter Assay

HEK293T cells were transfected with Gli-luc, TOPFLASH-luc, and Hes-1-conjugated firefly plasmid, together with Renilla-luc (pRL-TK) plasmid, using X-treme GENE 9 DNA transfection reagent (Roche, Werk Penzberg, Germany). At 12 h after transfection, cells were treated with crude *Niebla* sp. acetone extract, tumidulin, or 0.01% DMSO and incubated for 48 h at 37 °C under 5% CO_2_. Normalized luciferase activity was obtained against Renilla activity to determine the transfection efficiency using a Dual-Luciferase reporter assay system (Promega, Madison, WI, USA).

### 4.6. Spheroid Assay

Cells were trypsinized then rinsed with N2-supplemented DMEM/F12 (Invitrogen, Carlsbad, CA, USA) having human recombinant epidermal growth factor (hrEGF; 20 ng/mL; Biovision, Atlanta, GA, USA) and human basic fibroblast growth factor (hbFGF; 10 ng/mL; Invitrogen) and then seeded at a density of 1 × 10^4^ cells/well in ultra-low attachment 24-well plates (Corning Inc., Corning, NY, USA). After 14 days of culture, spheres were quantitated by inverted phase contrast microscopy (Nikon, Kawasaki, Japan). The IMT iSolution software (IMT iSolution Inc., Northampton, NJ, USA) was used to measure the pixel intensity of the sphere area from random microscope views in each plate. To measure the percentage area of spheres, normalization of the number of pixels in the sphere area was done by multiplying the given pixels by the pixel squares. Data are presented as the average of three independent experiments.

### 4.7. MTT Assay

Cells were seeded at a density of 2.5 × 10^3^ cells/well in 96-well plates, grown overnight, and then treated with the lichen acetone extracts or pure active fraction for 48 h. All the tested samples were dissolved in DMSO and diluted with DMEM to obtain indicated concentrations. The 3-(4,5-dimethylthiazol-2-yl)-2,5-diphenyltetrazolium bromide (MTT) was added and maintained for 4 h once treatment was completed. DMSO was added after removing the medium. Absorbance at 540 nm was determined using a microplate reader with Gen 5 (2.03.1) software (BioTek, Winooski, VT, USA).

### 4.8. Western Blotting

Cells treated with DMSO, *Niebla* sp. (1), or tumidulin for 48 h were washed twice with ice-cold phosphate-buffered saline (PBS) and lysed in lysis buffer [24]. Antibodies against ALDH1, CD44, CD133, Lgr5, Msi1, and α-tubulin were used as previously described [17]. Antibodies against Gli1 (sc-20687; SANTA CRUZ, Dallas, TX, USA), Gli2 (sc-271786; SANTA CRUZ), Smoothened (SMO; ab72130; Abcam, Cambridge, MA, USA), and β-Actin (sc-47778; SANTA CRUZ) were detected with horseradish peroxidase-conjugated secondary antibody (Thermo Fisher Scientific, Waltham, MA, USA) using an Immobilon Western Chemiluminescent HRP Substrate Kit (Merck Millipore, Billerica, MA, USA) and luminescence imaging on an Image Quant LAS 4000 mini. Bands were measured using Multi-Gauge 3.0 software, and the relative density was calculated based on the density of the β-actin bands in each sample. Values are expressed as arbitrary densitometric units corresponding to signal intensity.

### 4.9. Quantitative Reverse-Transcription PCR (qRT-PCR)

All quantitative reverse-transcription PCR (qRT-PCR) assays were performed as previously described [25]. Briefly, RNA (1 mg) isolated from CSC221 cells treated with DMSO, *Niebla* sp. (1), or tumidulin for 48 h using RNAiso Plus (TaKaRa, Otsu, Shiga 520-2193, Japan) was converted to cDNA using a M-MLV Reverse Transcriptase Kit (Invitrogen) and SYBR green (Enzynomics, Seoul, Korea). Primers used for qRT-PCR were as follows: ALDH1 (forward) 5′-tgttagctcatgccgacttg-3′ and (reverse) 5′-ttcttagcccgctcaacact-3′; Msi-1 (forward) 5′-accaagagatccaggggttt-3′ and (reverse) 5′-tcgttcgagtcaccatcttg-3′; Lgr5 (forward) 5′-ctcttcctcaaaccgtctgc-3′ and (reverse) 5′-gatcggaggctaagcaactg-3′; CD44 (forward) 5′-tgccgctttgcaggtgtat-3′ and (reverse) 5′-ggcctccgtccgagaga-3′; CD133 (forward) 5′-ggacccattggcattctc-3′ and (reverse) 5′-caggacacagcatagaataatc-3′; GAPDH (forward) 5′-atcaccatcttccaggagcga-3′ and (reverse) 5′-agttgtcatggatgaccttggc-3′. All reactions and analyses were performed using CFX (Bio-Rad, Hercules, CA, USA).

### 4.10. Statistical Analysis

All experiments were assayed in triplicate (*n* = 3). Results are reported as mean ± standard error of the mean (SEM). All statistical analyses were performed using the SPSS version 17 (IBM, Chicago, IL, USA). Treatment effects were determined using one-way ANOVA post-hoc analysis. The level of significance was set at *p* < 0.05.

## 5. Conclusions

In summary, tumidulin was isolated from Chilean lichen *Niebla* sp. (1; CH130494). Tumidulin exhibited significant inhibitory activity on spheroid formation in CRC cells and decreased the expression of CSC markers in CRC cells. The inhibitory activity of tumidulin against colorectal cancer stemness is through regulation of Hedgehog signaling pathways.

## Supplementary Materials

The following are available online.
