# Supplementary material for: Tumidulin, a Lichen Secondary Metabolite, Decreases the Stemness Potential of Colorectal Cancer Cells
Source: Molecules. 2018 Nov 14;23(11):2968. doi: 10.3390/molecules23112968 (PMC6278574; doi:10.3390/molecules23112968)

Supplementary Material

Figure S1. Relative viability of NIH 3T3 cells treated with a 5 μg/ml acetone extracts of lichens for 48 h by MTT assay.

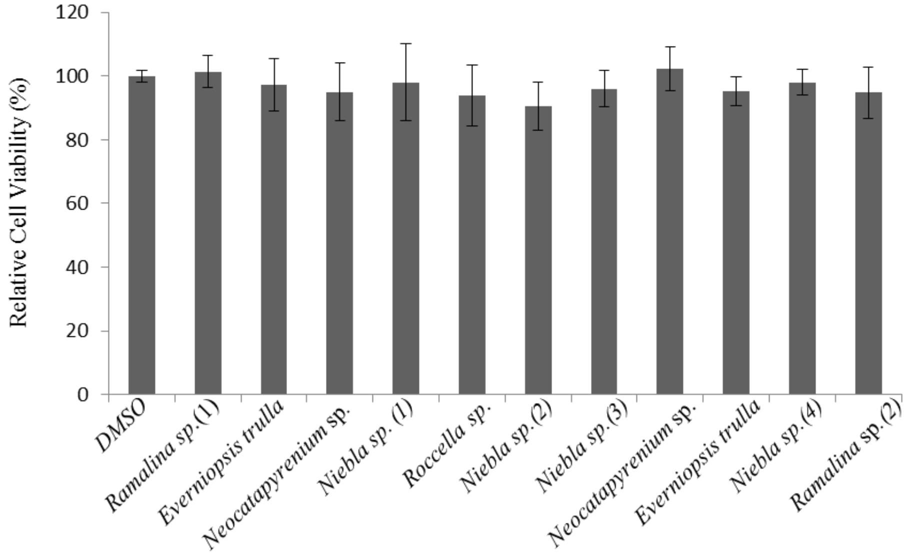

Figure S2. TLC results of lichen species

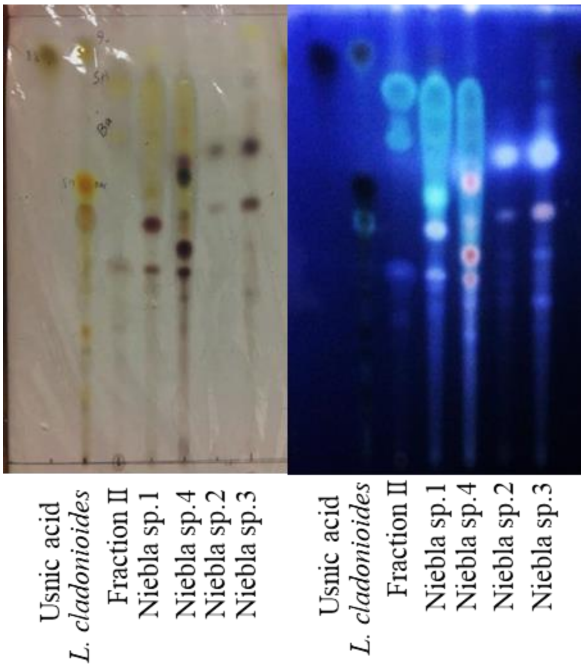

Figure S3. LC-MS spectrum of tumidulin

SHIMADZU LabSolutions **Analysis Report**

<Sample Information>

Sample Name	: Unknown Sample00
Sample ID	: UNK-0004
Data Filename	: SB-FH-55 20180612.lcd
Method Filename	: 15min, 254nm(ACN 10 to 90).lcm
Batch Filename	: INJECTION(no shut down).lcb
Vial #	: 1-3
Injection Volume	: 10 uL
Date Acquired	: 2018-06-12 오후 3:16:58
Date Processed	: 2018-06-12 오후 3:32:00
Sample Type	: Unknown
Acquired by	: System Administrator
Processed by	: System Administrator

<Chromatogram>

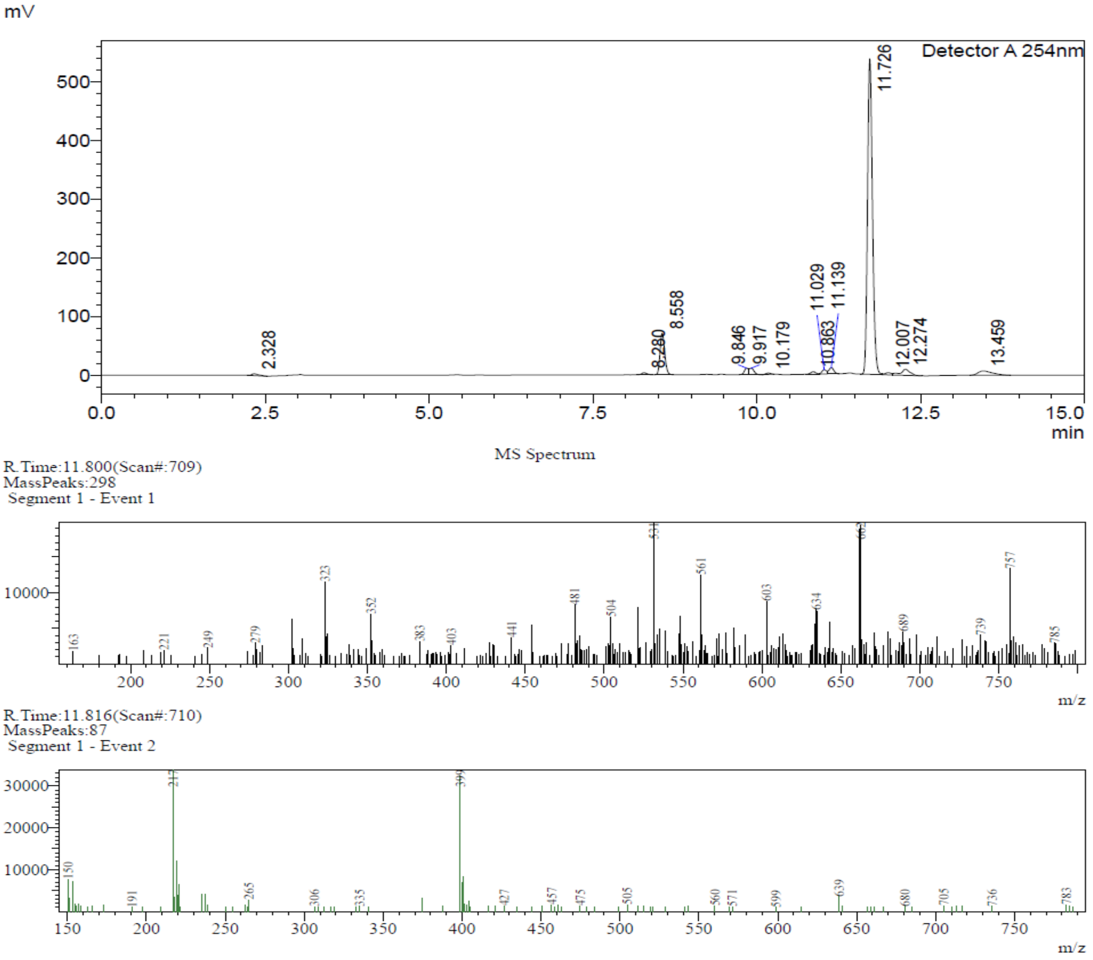

Figure S4. ^{1}H-NMR spectrum of tumidulin

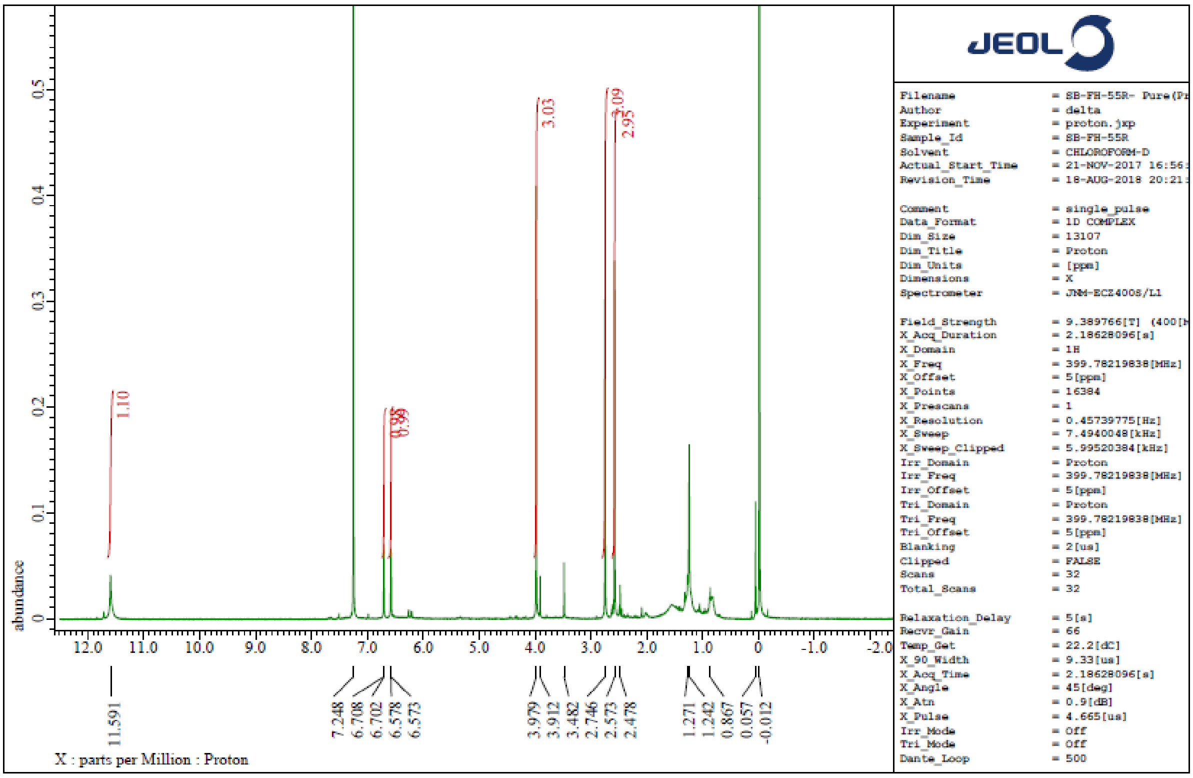

^{1}H-NMR(400 MHz, $CDCl_3$): 2.573(s, 3H), 2.746(s, 3H), 3.979(s, 3H), 6.573(s, 1H), 6.702(s, 1H), 11.591(s, OH)

Figure S5. ^{13}C -NMR spectrum of tumidulin.

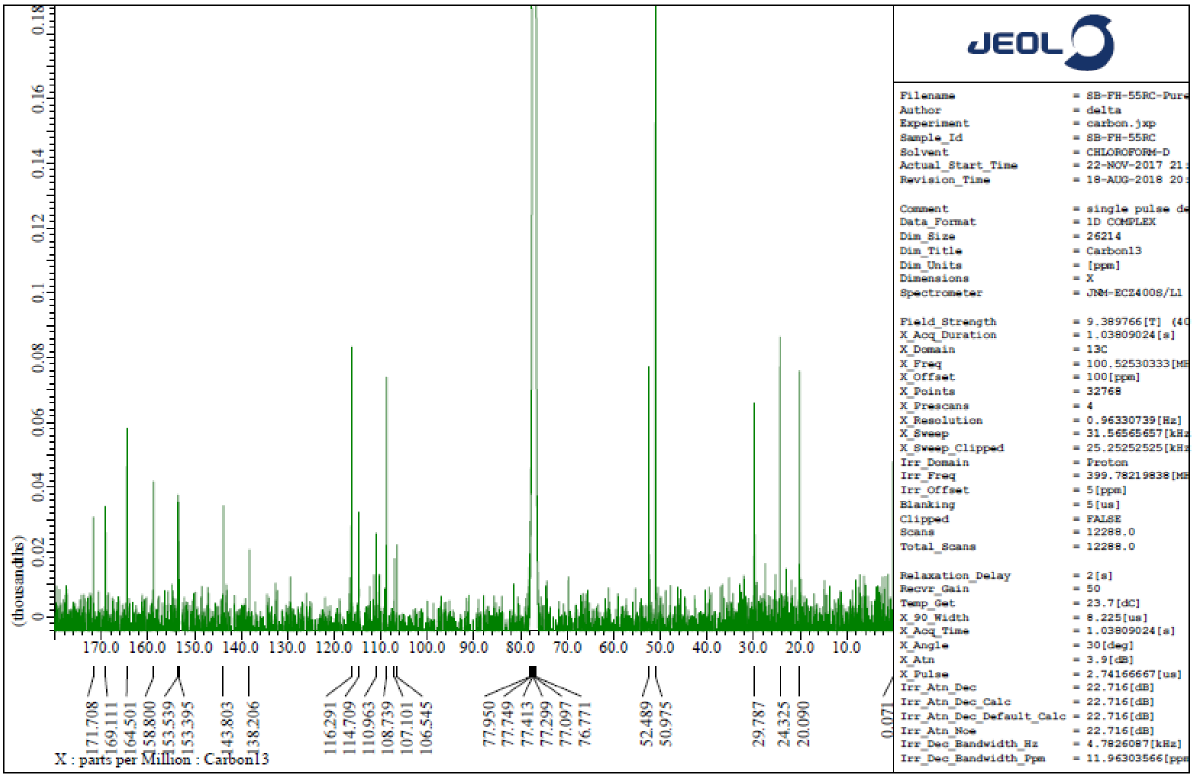

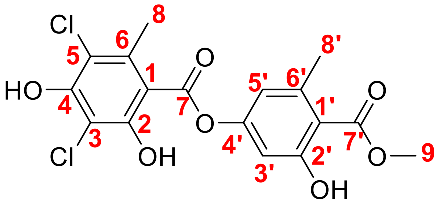

^{13}C-NMR(400 MHz, $CDCl_3$): C-1:110.963, C-2: 158.800, C-3: 107.101, C-4: 153.539, C-5: 116.291, C-6: 138.206, C-7: 169.111, C-8: 20.090, C-9: 52.489, C-1’: 108.739, C-2’: 164.501, C-3’: 106.545, C-4’: 153.395, C-5’: 114.709, C-6’: 143.803, C-7’: 171.708, C-8’: 24.325

Figure S6. ^{1}H-NMR spectrum of an acetone extract of *Niebla* sp. (1).

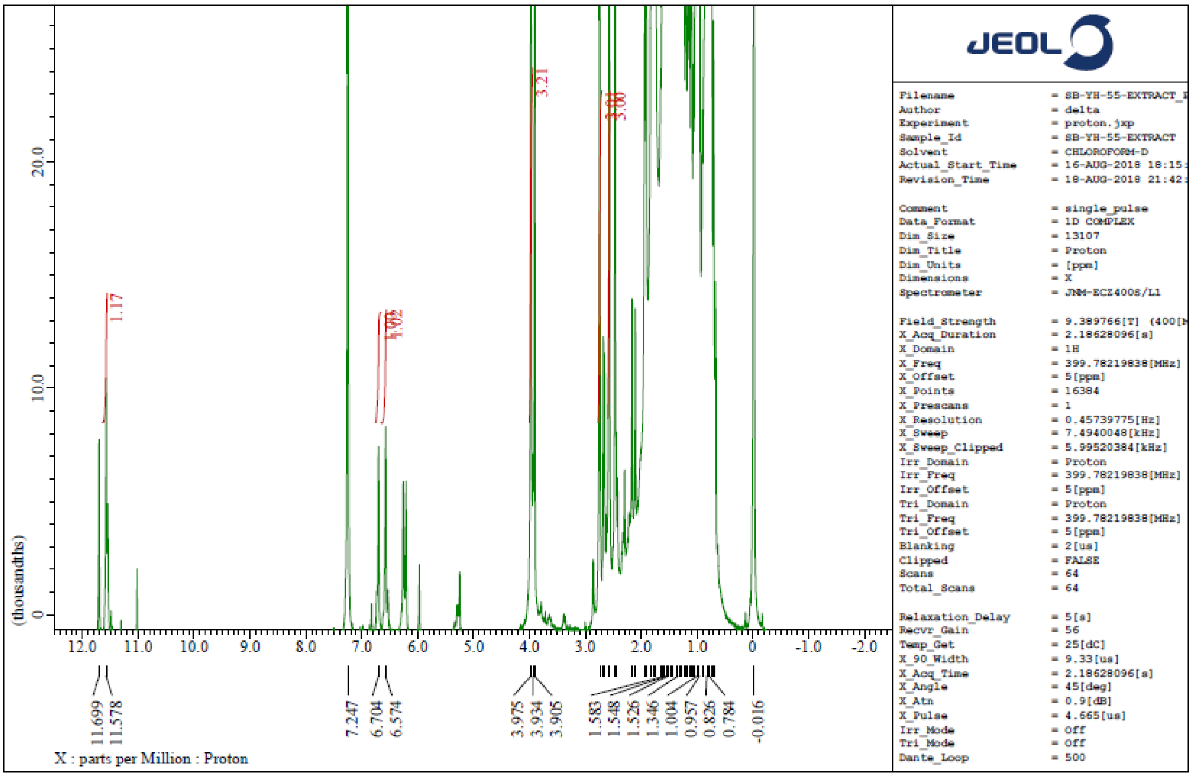

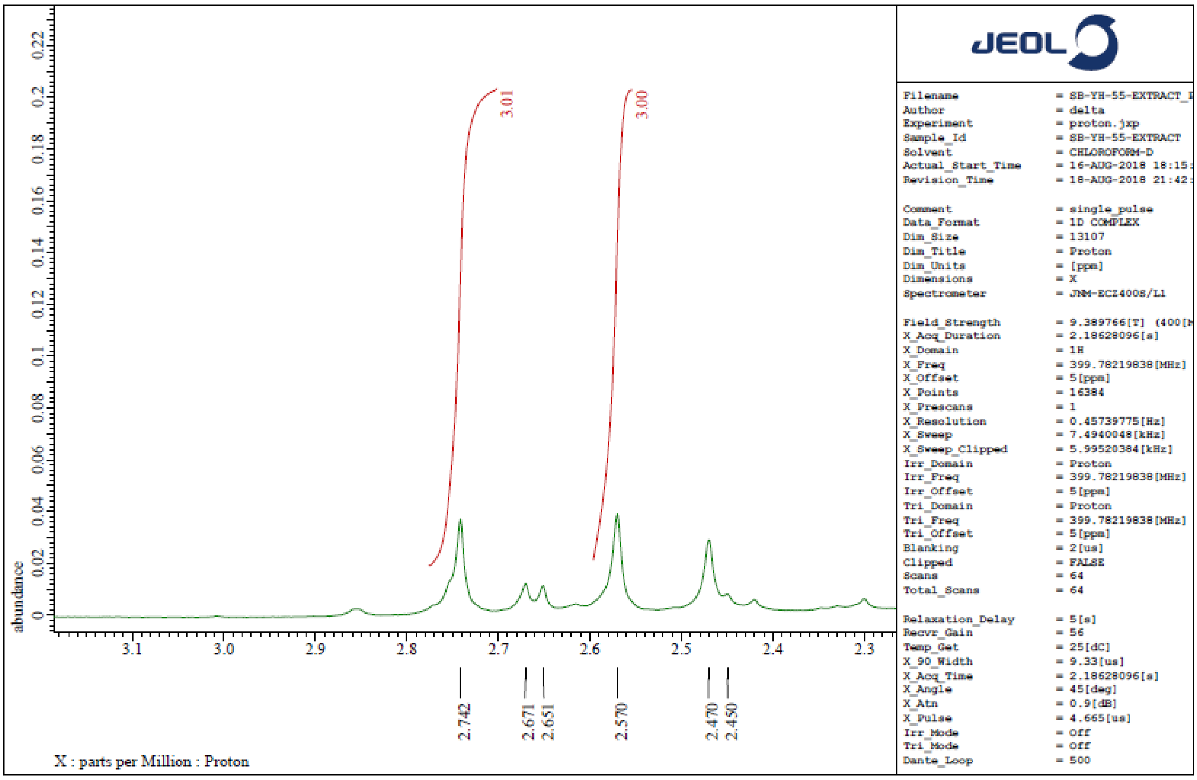

^{1}H-NMR(400 MHz, $CDCl_3$): 2.570(s, 3H), 2.742s, 3H), 3.975(s, 3H), 6.574(s, 1H), 6.704(s, 1H), 11.578(s, OH)

Figure S7. ^{13}C -NMR spectrum of an acetone extract of *Niebla* sp. (1).

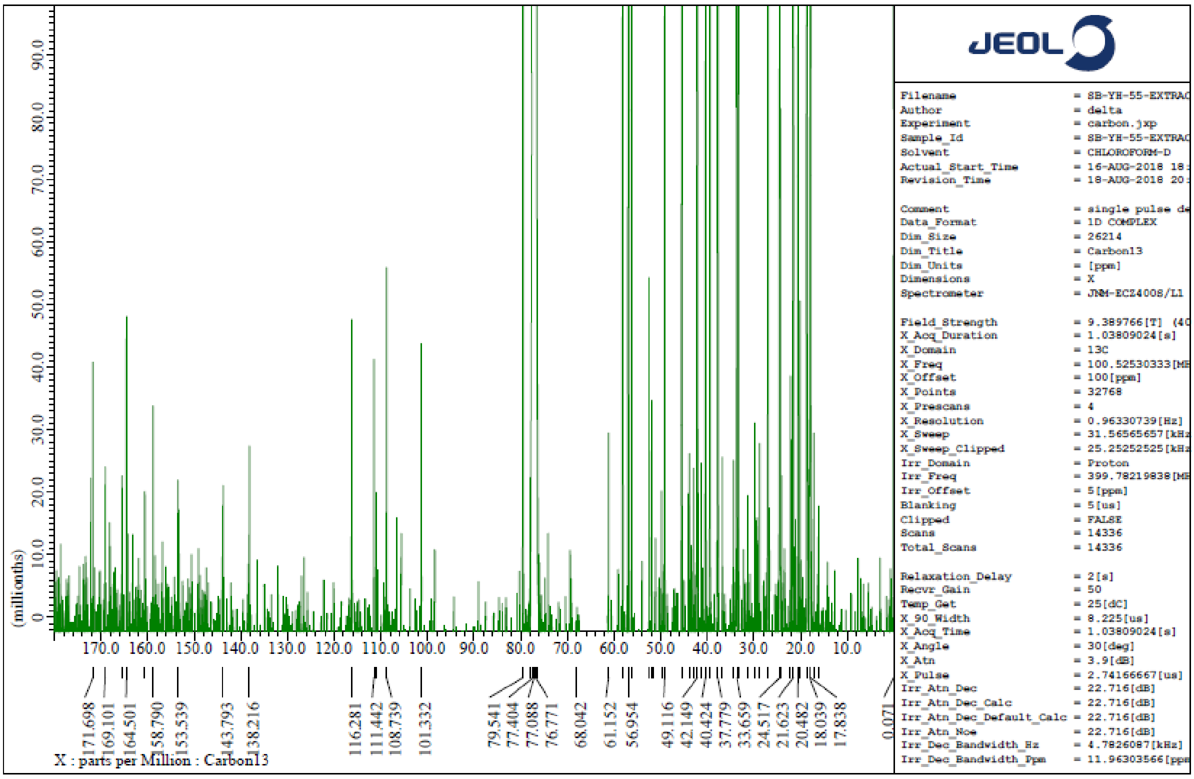

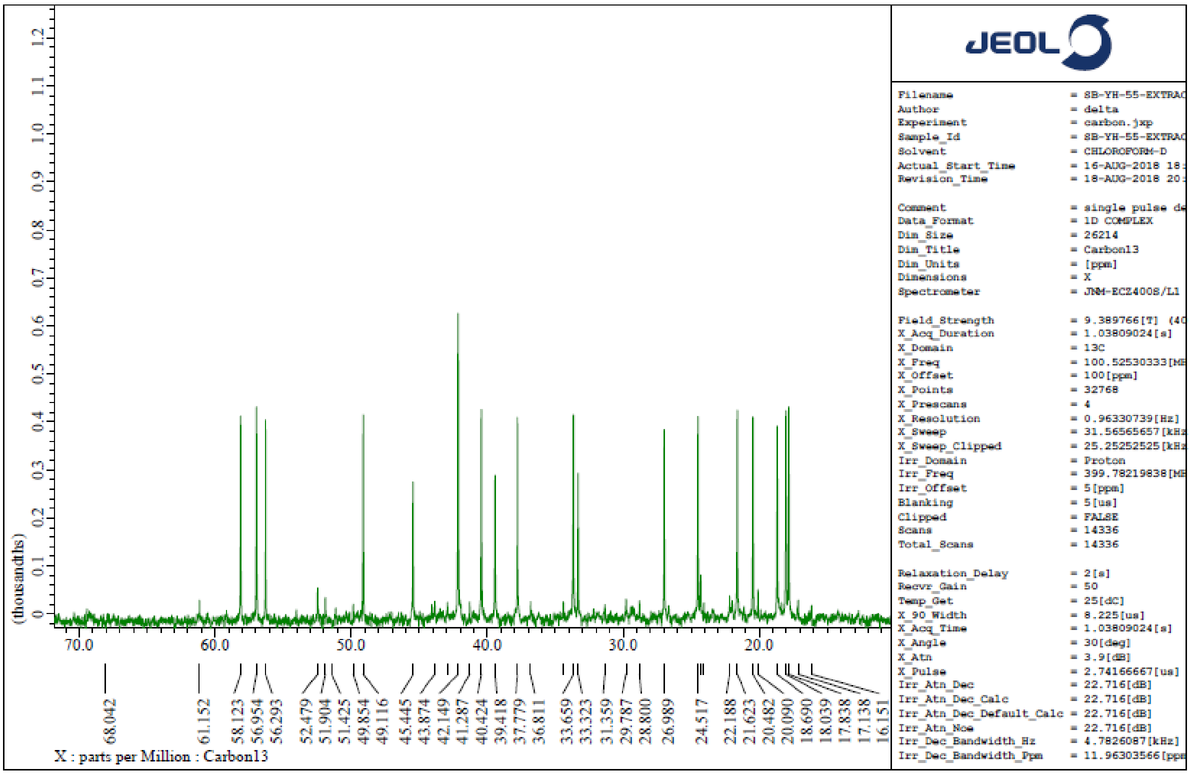

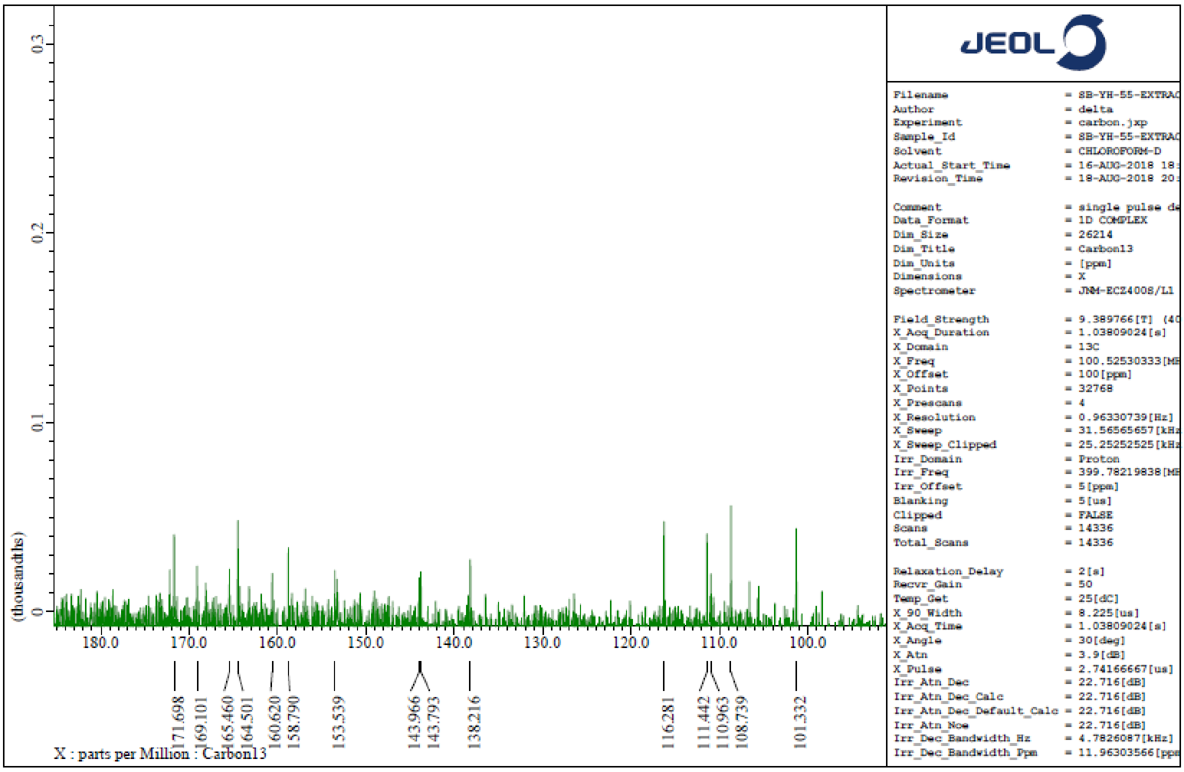

^{13}C-NMR(400 MHz, $CDCl_3$): C-1:110.963, C-2: 158.790, C-3: 101.332, C-4: 153.539, C-5: 116.281, C-6: 138.216, C-7: 169.101, C-8: 20.482, C-9: 52.479, C-1’: 108.739, C-2’: 164.501, C-3’: 106.526, C-4’: 153.347, C-5’: 111.441, C-6’: 143.793, C-7’: 171.608, C-8’: 24.316

Figure S8. Relative viability of CRC cells treated with a 5 μg/ml acetone extracts of sp. (1) and 12.5 μM tumidulin for 48 h by MTT assay.

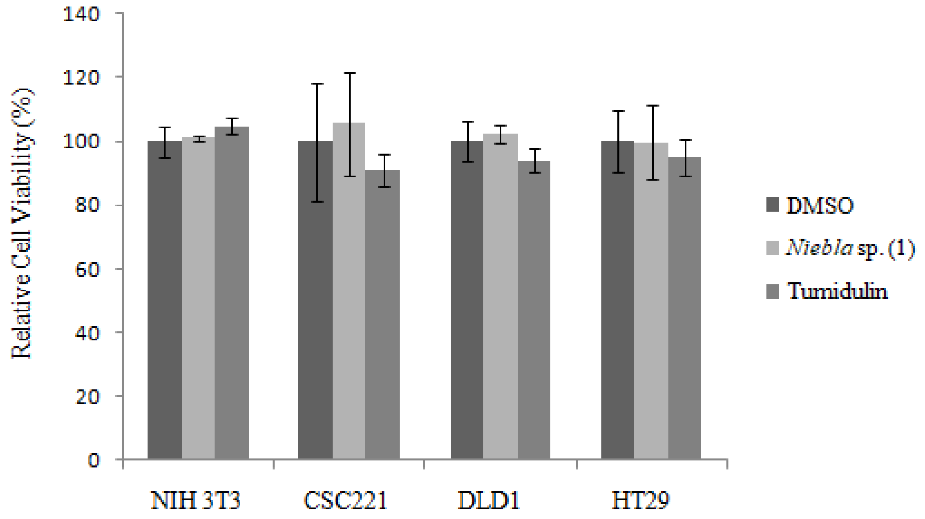

Supplement: Supplementary file 1 [file molecules-23-02968-s001.pdf]
